# P-1013. Desperate Times Call for Desperate Measures, but is it Sustainable? The Nightmare on Hospital-Onset Clostridium Difficile Infection

**DOI:** 10.1093/ofid/ofaf695.1210

**Published:** 2026-01-11

**Authors:** Maria Akiki, Dora E Wiskirchen, Jessica Abrantes-Figueiredo

**Affiliations:** University of Connecticut, Hartford, CT; Saint Francis Hospital and Medical Center, Hartford, Connecticut; Saint Francis Hospital, Hartford, Connecticut

## Abstract

**Background:**

Hospital-onset *Clostridioides difficile* infection (CDI) is a significant cause of hospital-acquired infections globally. Overuse and inappropriate testing often lead to false-positive cases classified under the National Healthcare Safety Network criteria for hospital-onset CDI (HO-CDI), despite not representing true infections, leading to unnecessary treatment, prolonged isolation, and increased healthcare costs. This quality improvement project evaluates whether prospective audit and feedback on testing, combined with a diagnostic algorithm, can reduce inappropriate testing and HO-CDI cases.

**Figure:**
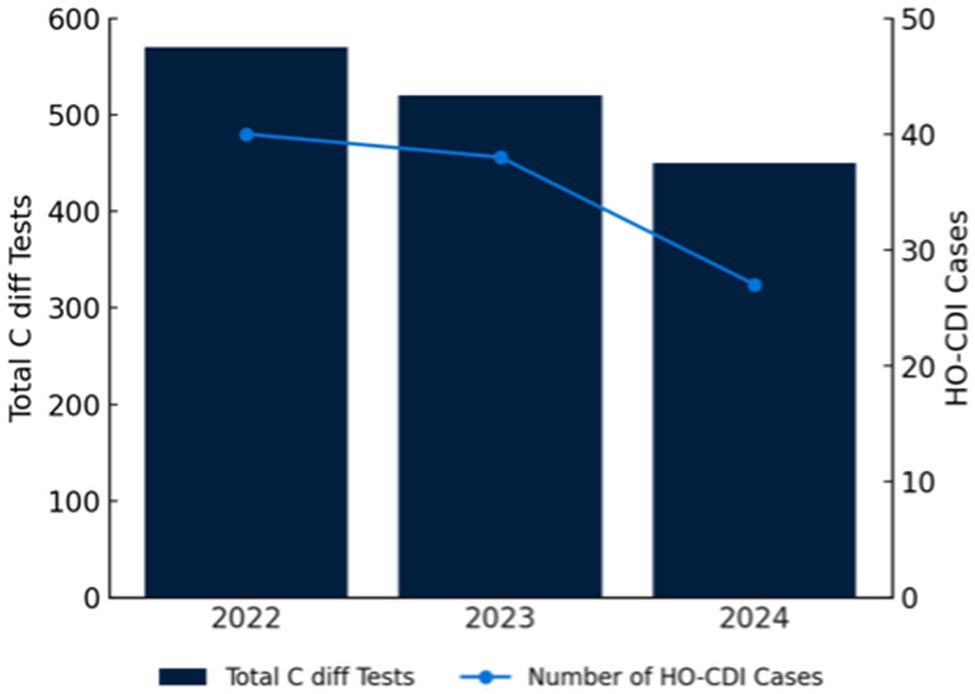
Annual Inpatient Clostridium Difficile Testing and Hospital-Onset Clostridium Difficile Infection Cases Annual inpatient Clostridioides difficile testing and hospital-onset Clostridioides difficile infection cases from 2022 to 2024. Total tests (bars) and hospital-onset infection cases (line) both declined, with the most significant reduction observed in 2024 following the diagnostic stewardship intervention.

**Figure:**
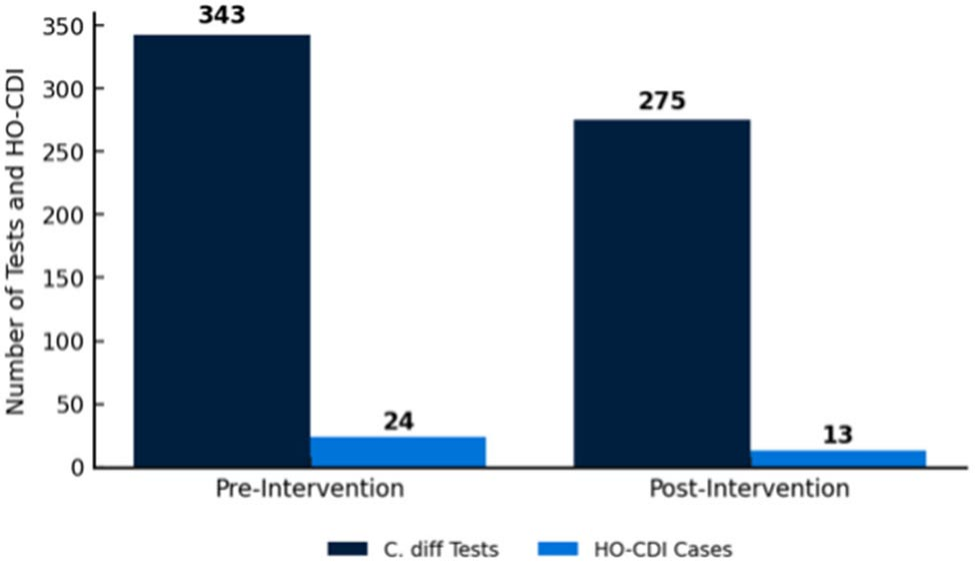
Clostridium Difficile Testing and Hospital-Onset Clostridium Difficile Infection Cases Before and After the Intervention Comparison of Clostridioides difficile testing and hospital-onset Clostridioides difficile infection cases before and after the implementation of a diagnostic stewardship intervention. The number of tests and hospital-onset infection cases both decreased following the intervention, indicating more appropriate test utilization and lower infection rates.

**Methods:**

An intervention began in May 2024, involving microbiology laboratory technicians contacting the chief of infectious disease to review the appropriateness of *Clostridium difficile* (C. diff) testing for samples collected 48-72 hours post-admission. Additionally, a hospital-wide diagnostic algorithm was introduced to guide appropriate testing. Total inpatient C. diff tests before and after the intervention were compared. Percentage reductions and paired t-tests (p < 0.05) were used to evaluate changes. The project was IRB-exempt.

**Results:**

C. diff testing declined steadily, with a 10.36% drop from 2022 to 2023 (p = 0.1476) and a 14.07% drop from 2023 to 2024 (p = 0.0809). HO-CDI cases fell by 2.63% from 2022 to 2023 (p = 0.6742) and 27.03% from 2023 to 2024 (p = 0.0001). After the intervention, testing decreased by 19.60% (p = 0.0581) and hospital-onset CDI by 48.48% (p = 0.0052) compared to pre-intervention. CDI cases as a percentage of tests dropped from 9.51% to 6.09% (35.93% reduction, p = 0.9057), while hospital admissions remained stable.

**Conclusion:**

The sharper decline in C. diff testing from 2023 to 2024 (14.07% vs. 10.36%) suggests the intervention helped reduce unnecessary testing. Post-intervention, HO-CDI fell by 48.48% (p = 0.0052), while testing dropped by 19.60%, indicating improved test appropriateness rather than under-diagnosis. These results support existing evidence that audit and feedback can reduce over-testing and improve CDI outcomes by aligning testing with guidelines, minimizing unnecessary antibiotics, and isolation. Future research should assess sustaining improvements and refining stewardship to ease reliance on a testing gatekeeper.

**Disclosures:**

All Authors: No reported disclosures

